# Effects of Citalopram on Sutural and Calvarial Cell Processes

**DOI:** 10.1371/journal.pone.0139719

**Published:** 2015-10-02

**Authors:** Emily Durham, Serena Jen, Lin Wang, Joseph Nasworthy, Mohammed Elsalanty, Seth Weinberg, Jack Yu, James Cray

**Affiliations:** 1 Departments of Oral Health Sciences, Medical University of South Carolina, Charleston, South Carolina, United States of America; 2 School of Medicine, Georgia Regents University, Augusta, Georgia, United States of America; 3 Institute for Plastic Surgery, Shanghai Jiao Tong University, Shanghai, China; 4 Department of Oral Biology, Georgia Regents University, Augusta, Georgia, United States of America; 5 Department of Cellular Biology and Anatomy, Georgia Regents University, Augusta, Georgia, United States of America; 6 Department of Oral Maxillofacial Surgery, Georgia Regents University, Augusta, Georgia, United States of America; 7 Institute for Regenerative and Reparative Medicine, Georgia Regents University, Augusta, Georgia, United States of America; 8 Center for Craniofacial and Dental Genetics, School of Dental Medicine, University of Pittsburgh, Pittsburgh, Pennsylvania, United States of America; 9 Department of Oral Biology, School of Dental Medicine, University of Pittsburgh, Pittsburgh, Pennsylvania, United States of America; 10 Department of Surgery, Division of Plastic Surgery, Georgia Regents University, Augusta, Georgia, United States of America; Public Health Research Institute at RBHS, UNITED STATES

## Abstract

The use of selective serotonin reuptake inhibitors (SSRIs) for the treatment of depression during pregnancy is suggested to increase the incidence of craniofacial abnormalities including craniosynostosis. Little is known about this mechanism, however based on previous data we propose a mechanism that affects cell cycle. Excessive proliferation, and reduction in apoptosis may lead to hyperplasia within the suture that may allow for differentiation, bony infiltration, and fusion. Here we utilized *in vivo* and *in vitro* analysis to investigate this proposed phenomenon. For *in vivo* analysis we used C57BL–6 wild-type breeders treated with a clinical dose of citalopram during the third trimester of pregnancy to produce litters exposed to the SSRI citalopram *in utero*. At post-natal day 15 sutures were harvested from resulting pups and subjected to histomorphometric analysis for proliferation (PCNA) and apoptosis (TUNEL). For *in vitro* studies, we used mouse calvarial pre-osteoblast cells (MC3T3-E1) to assess proliferation (MTS), apoptosis (Caspase 3/7-activity), and gene expression after exposure to titrated doses of citalopram. *In vivo* analysis for PCNA suggested segregation of effect by location, with the sagittal suture, showing a statistically significant increase in proliferative response. The coronal suture was not similarly affected, however there was a decrease in apoptotic activity at the dural edge as compared to the periosteal edge. No differences in apoptosis by suture or area due to SSRI exposure were observed. *In vitro* results suggest citalopram exposure increased proliferation and proliferative gene expression, and decreased apoptosis of the MC3T3-E1 cells. Decreased apoptosis was not confirmed *in vivo* however, an increase in proliferation without a concomitant increase in apoptosis is still defined as hyperplasia. Thus prenatal SSRI exposure may exert a negative effect on post-natal growth through a hyperplasia effect at the cranial growth sites perhaps leading to clinically significant craniofacial abnormalities.

## Introduction

Research has suggested selective serotonin reuptake inhibitors (SSRIs) used to treat depression in pregnant women may increase the risk of birth defects, specifically those of the craniofacial skeleton [1–7]. These data remain controversial [8–14] and there is lack of agreement concerning safety, trimester of use, drug specificity and dose, causation and mechanism of effect. Still, life time risk of major depression among women approaches 25% and is highest during their childbearing years, resulting in high use of antidepressants during pregnancy. In the United States, it is estimated that 7% to 13% of pregnant women have consumed antidepressants and numbers are rising as are concerns for the health outcomes of newborns [15, 16]. Along these lines the once heavily prescribed SSRI, paroxetine (Paxil) has been re-classified for pregnancy risk from class C to class D, indicating its potential risks to the developing fetus, specifically for cardiac anomalies [17].

There are now clinical reports linking SSRI use and the developmental disruption of cranial vault size, shape and possible association with craniosynostosis [4, 5]. Such results have previously been observed in preclinical models [18–20]. Craniosynostosis is defined as the premature fusion of one or more of the fibrous joints between the major bones of the skull known as cranial sutures [21, 22]. Worldwide incidence of craniosynostosis is 1 in every 2100 to 2500 live births with commonly reported comorbidities of mental retardation, deafness, optic nerve compression, altered cranial volume, midface growth discrepancies and increased intracranial pressure. The etiology of craniosynostosis is poorly understood but incidences can occur due to targeted gene mutations (FGF, Twist, MSX2) or environmental factors but in the majority of cases causation is unknown [21–23]. Our group has previously investigated the effects of one SSRI, citalopram (*Celexa*) on the developing murine craniofacial skeleton. Utilizing *in utero* exposures to a wild-type model we confirmed *in vivo* effects in the offspring similar to that previously reported in the aforementioned preclinical and clinical studies including altered cranial vault shape, decreased cranial growth, and craniofacial anomalies including suture synostosis [24].

There are several hypotheses for the pathogenesis of craniosynostosis focused on cellular imbalances including aplasia (suture agenesis), dysplasia (change in cell phenotype, essentially differentiation) and hyperplasia due to an imbalance between the processes of cell proliferation and apoptosis of the cells that comprise the suture mesenchyme [21, 25–31]. In a normal developing skull, a balance exists between cell proliferation and apoptosis, whereas, in the craniosynostotic skull, cell proliferation is believed to be a dominant and disruptive force. This imbalance likely leads to later differentiation of cells in the suture allowing for bony infiltration and fusion. In our previous research using murine calvarial cells we observed increased markers of osteoblastogenesis as well as alteration in overall cell proliferation after citalopram exposure [24]. Though poorly understood, there is now evidence to suggest that serotonin (SSRIs use results in increased production of brain serotonin) may play a significant role in early craniofacial development, including brain development, neural crest cell migration and development of the structures of the head and face as well as normal bone physiology [2, 5, 32–34]. Given the frequency of SSRI use during pregnancy it is imperative that any potential teratogenic effects be subject to continued investigation for these drugs. Based on previous data, here we sought to test the hypothesis that exposure to SSRIs would alter the proliferation and apoptosis of perisutural cells at the cellular and transcription levels.

## Materials and Methods

### SSRI Treated Perinates

We previously reported on the effects of *in utero* citalopram exposure on postnatal skull morphology [24]. To investigate *in vivo* effects of SSRI treatment on proliferation and apoptosis at the cranial growth sites, representative samples were utilized here from our sample of exposed (n = 24) and unexposed (n = 26) 15 day old C57BL6 mice. As previously described, individually housed pregnant C57BL6 dams (Jackson Laboratories, Bar Harbor, ME) were administered a dose of approximately 500 micrograms per day in drinking water of citalopram from embryonic day (E)13 to E20 (roughly the third trimester). Dosage was identified as midrange for preclinical studies in murine models and were replaced as needed to provide ad labium access [35–37]. Resulting offspring were allowed to grow to postnatal day 15 when they were sacrificed via carbon dioxide asphyxiation and subsequent cervical dislocation and decapitation. Calvaria were harvested then isolated for use in these studies. The Animal Use Protocol was reviewed and approved by the Georgia Regents University Institutional Animal Care and Use Committee, 2012–0498. All breeding procedures were carried out in an Association for Assessment and Accreditation of Laboratory Animal Care International accredited facility where all husbandry and related services are provided by the Division of Laboratory Animal Resources.

### Histomorphometrics

Randomly selected calvarial samples per group (control (unexposed) and exposed n = 3) were fixed in 3.7% formaldehyde for 48 hours then were decalcified in 0.25M EDTA at pH7.4 for 10 days with changes of the EDTA every 3 days. The sutures were isolated from the top of the calvaria and were washed, dehydrated in a graded series of ethyl alcohol (70–100%), cleared in xylene, and embedded in paraffin. Prior to embedding, samples were cut in half posterior and parallel to the coronal suture and the front half of the calvaria was again bisected along the sagittal suture to allow for embedding in an orientation that allowed for cutting through both sutures at 8μm using a rotary microtome prior to mounting on Super Frost Plus glass slides for histology.

Serial sections were subjected to immunohistochemistry for Proliferating Cell Nuclear Antigen (PCNA), a marker associated with cell proliferation. Briefly, sections were depraffinized and rehydrated to water. Endogenous peroxidase was blocked with 3% hydrogen peroxide in methanol, and then 1% Goat Serum was used to block non-specific binding. Sections were then incubated with PCNA primary antibody (AbCam, Cambridge, MA, USA, ab18197) diluted 1 to 3000 in Goat Serum for 1 hour at room temperature. After three washes with Phosphate-Buffered Saline (PBS) sections were incubated with Goat Anti-Rabbit Secondary Antibody conjugated for HRP (AbCam, ab6721) for 1 hour at room temperature and were subsequently washed again with PBS three times. Finally, sections were exposed to 3,3’-diaminobenzidine (DAB) (Vectror Laboratories, Bulingame, CA, USA) for 3 minutes, the reaction was stopped with water, and the sections were dehydrated, cleared, and mounted with permount (Fisher Scientific, Waltham, MA, USA). Sections serial to those stained with PCNA served as secondary antibody only controls and were counterstained for 30 seconds with Harris Hematoxylin diluted 1:10 in water after the DAB reaction was stopped. Slides were then imaged with a VWR microscope equipped with a moticam digital camera and imaging software (VWR, Radnor, PA, USA).

Terminal deoxynucleotidyl tranferase dUTP nick end labeling (TUNEL), a method for detecting DNA fragmentation, was performed according to the manufacturer protocol (Roche, Indianapolis, IN, USA, 11 684 795 910). Briefly, sections were deparaffinized and rehydrated, and then subjected to epitope retrieval via a 1 minute microwave incubation with 0.1mM Sodium Citrate Buffer (10mM Sodium Citrate 0.5% Tween 20 pH 6.0). After cooling in water and washing in PBS the slides were incubated in Tris-HCl buffer (0.1M Tris HCl ph 7.5 3% Bovine Serum Albumin 20% Fetal Bovine Serum), then washed tree times in PBS and incubated with activated TUNEL reaction staining solution for 1 hour at 37 degrees in the dark. After incubation, the slides were washed with PBS and mounted using Vectashield 4',6-diamidino-2-phenylindole (DAPI) (Vector Laboratories, Bulingame, CA, USA) mounting media. Slides were then imaged with an Olympus BX61 microscope equipped with an Olympus DP72 8-bit RGB camera and Cellsens imaging software (Olympus, Waltham, MA, USA).

Image analysis was performed using Image J Software [38]. Average background threshold was determined by selecting 3 areas per control (secondary only, or inactive TUNEL reaction staining solution) image and determining the threshold at which no pixels were able to be identified. This threshold was then applied to all images taken of either staining technique. The sutural area was isolated as well as the osteogenic front for three sections (at least 30μm apart) for each suture for each group (control (unexposed) and exposed n = 3). For PCNA total cell count was identified via hematoxylin stain in a section serial to the section stained for PCNA. Cells positive for PCNA were then identified as a percent of the total cells counted. For localization of staining (periosteal, midsutural, dural and osteogenic front) the suture was measured from dura to periosteum and that length was divided by 3 to determine the size of the localized areas. For TUNEL staining total cell count was identified via a DAPI nuclear counterstain. Photos were taken of the same section using different filters to allow for the FITC positive TUNEL staining to be compared to the total cell count from the DAPI staining. Merged images of the different excitation channels were also created.

### Cell Culture and Characterization

Previous data has demonstrated a significant effect of the SSRI citalopram on osteogenic response and relevant gene expression on mouse calvarial pre-osteoblasts [24]. The MC3T3-E1 murine calvaria osteoprogenitor cells are a stock immortalized cell line (American Type Culture Collection (ATCC), Manassas, VA, USA). Here we use those same cells to determine direct changes to proliferation or apoptosis after citalopram exposure. Cells were cultured in alpha minimum Eagles medium (αMEM, Lonza, Allendale, NJ, USA) supplemented with 1% penicillin/streptomycin (pen/strep, Life Technologies, Grand Island, NY, USA), 10% fetal bovine serum (FBS, Atlanta Biologicals, Flowery Branch, GA) and Amphotericin B (Lonza).For control data, cells were cultured for 3 or 7 days with standard alpha proliferation media. For SSRI treatments, media was supplemented with citalopram eluted to serially diluted doses between 10^−4^ mol through 10^−10^ mol to achieve a dose response curve.

The effects of SSRI exposures on metabolic markers of cell proliferation and apoptosis were assessed via MTS [39] and Apo-ONE Homogeneous Caspase–3/7 (Promega, Madison, WI, USA) per manufacturer instructions. Cells were seeded in triplicate into 96-well plates at a density of approximately 4,000 cells/well and treated for 3 or 7 days. For MTS assay after 3 or 7 days of treatment, cells were incubated for one hour with 20μl/well of CellTiter96 Aqueous One solution. The absorbance at 490nm was recorded with a 96-well plate reader (Biotek, Synergy H1 Mono, Winooski, VT, USA). Our sample size was n = 3 per condition. For Caspase 3/7 apoptosis assay after 3 or 7 days of treatment, cells were incubated with 100 μl/well substrate/buffer solution (1:100). Contents were mixed for 30 seconds at 300 rpm and incubated at room temperature for one hour. Fluorescence was measured using a 96-well plate reader (Biotek) with excitation at 485 and emission at 530 nm. Our sample size was n = 3 per condition.

### Quantitative Real Time Polymerase Chain Reaction (qrt-PCR)

Quantitative real time polymerase chain reactions (qrt-PCRs) were run using a one-step kit for cDNA synthesis and gene expression (TaqMan® RNA-to-Ct™ 1-Step Kit, Life Technologies, Thermo-Fisher Scientific, Walltham, MA, USA) following manufacturer instructions. A master mix was made from nuclease free water, rt-mix, enzyme mix and commercially prepared probe/primer sets (Taqman Gene Expression Assays, Table 1). A total of 50ug of RNA from murine calvaria osteoprogenitor cells was added to the master mix for each reaction (run in duplicate) for each gene product for each condition time (3 or 7 day) by treatment (media only control or media supplemented with 10^−4^ mol/L citalopram). Data were normalized to 18S ribosomal RNA expression by ΔCT. Quantitative data were compared for gene expression change due to treatment by ΔΔCT methodology. Genes of interest were both markers associated with osteoblast progenitor proliferation, *Ki67*, *Ccnd1*, *Jun* and apoptosis *Bax*, *Casp3*, and *Bcl2*.

**Table 1:** Quantitative qRT-PCR TaqMan Probe/Primer Set Data.

*Gene Symbol*	*Gene Name*	*Primer Sequence*	*Context Sequence*
Mki67	Marker of Proliferation Ki67	Mm01278617_m1	GACAATCATCAAGGAACGGCCCCAG
Ccnd1	Cyclin D1	Mm00432359_m1	CTCTGTGCCACAGATGTGAAGTTCA
Jun	Jun Proto-oncogene	Mm00495062_s1	GAACTGGGGAGGAGGGCTCAGGGGG
Bax	BCL Associated X Protein	Mm00432051_m1	CAAGACCAGGGTGGCTGGGAAGGCC
Casp3	Caspase 3	Mm01195085_m1	TCTACAGCACCTGGTTACTATTCCT
Bcl2	B-cell CLL/lymphoma 2	Mm00477631_m1	CGGAGGCTGGGATGCCTTTGTGGAA
18S	18S ribosomal RNA	Mm03928990_g1	TACTTGGATAACTGTGGTAATTCTA

### Statistical Analysis

For ex-vivo PCNA and TUNEL analyses, data were utilized as a proportion of cells expressing the marker over the total number of cells per area of interest (periosteal, midsutural, dural and osteogenic front). Data was analyzed by three and two-way analysis of variance (ANOVA) to determine differences in PCNA and TUNEL activity due to exposure (none or citalopram), suture (differences between coronal or sagittal sutures), and area of the suture. All data was subjected to tests for normality (Shapiro-Wilk) and homogeneity of variances (Browne-Forsythe). Post-hoc analyses of intergroup differences were conducted using Bonferonni methodology. Differences were considered statistically significant if p<0.05.

For proliferation and apoptosis cellular assays, raw values were used to conduct two ANOVAs for treatment (untreated control and citalopram doses between 10^−4^ mol through 10^−10^ mol/liter) by assay time point, 3 or 7 days. All data was subjected to tests for normality (Shapiro-Wilk) and homogeneity of variances (Browne-Forsythe). Post-hoc analyses of intergroup differences were conducted using Bonferonni methodology. Differences were considered statistically significant if p<0.05. All statistical analyses were conducted using SPSS 22 (IBM, Armonk, NY, USA).

For qrt-PCR analyses were conducted following manufacturer description and published methodology plotting mean fold change and upper and low bounds confidence interval (+/- one standard deviation) (Applied Biosystems, Life Technologies). A fold change simply represents the percent increase or decrease in the amount of gene expression for a target subject to qrt-PCR and ΔΔCT methodology. A fold change that does not cross 1 or -1 can be considered to have some relevant biological alteration in expression level. In addition, we have followed statistical analysis as previously published to determine statistical differences for gene expression after citalopram treatment for target of interest by time of exposure [40]. Differences were considered statistically different if p≤0.05.

## Results

### Markers of Proliferation

**Fig 1:**
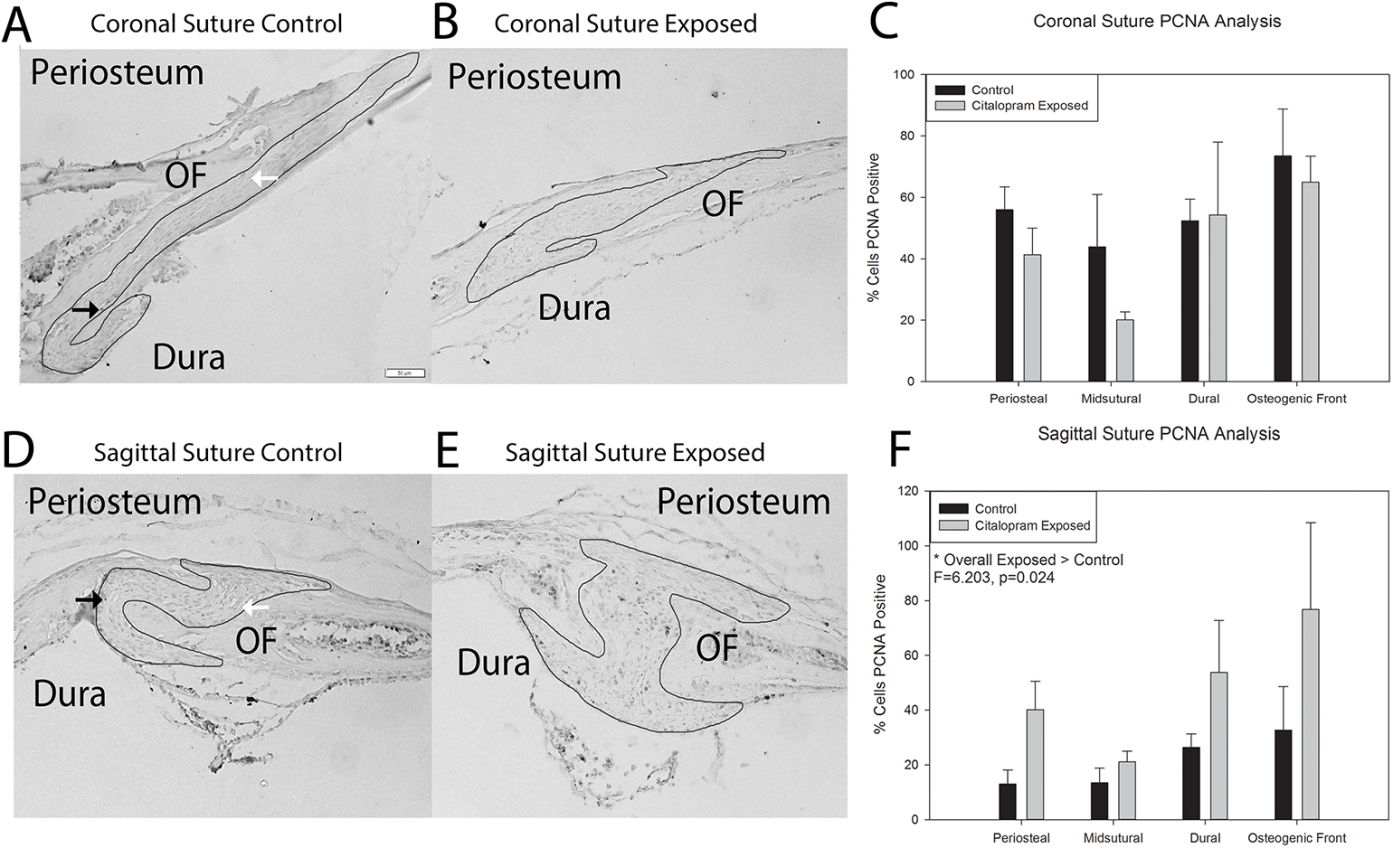
Histological markers of proliferation in control and citalopram exposed mouse cranial sutures. A & B. Coronal sutures from control (A) and citalopram exposed (B) 15 day old C57BL6 mice stained for Proliferating Cell Nuclear Antigen (PCNA) (black arrows = example of positive cell, white arrows = example of negative cells). Periosteum, dura and osteogenic front (OF) are as marked, and the sutural area has been outlined. C. Quantification of percent PCNA stain. PCNA activity shows slight decreases for all but dural associated areas, but no statistical differences. D & E. Sagittal sutures from control (D) and exposed (E) 15 day old C57BL6 mice stained for PCNA. F. Quantification of percent PCNA stain. Overall, PCNA activity increased in all areas assessed in citalopram exposed sagittal sutures.

Fig 1A, 1B, 1D and 1E demonstrates representative samples of Proliferating Cell Nuclear Antigen (PCNA, a marker of proliferation) staining for coronal and sagittal sutures from postnatal day 15 animals either unexposed (control) or exposed *in utero* to the SSRI citalopram. Qualitative assessment suggest increased PCNA staining for SSRI exposed animal sutures. Quantitative analysis was conducted for percent cells PCNA positive as a function of total cells present. A three-way analysis of variance was conducted to determine if the relationship for exposure (control or citalopram) by area of suture was the same for coronal and sagittal suture samples. Assumptions of normality and homogeneity of variance were met. Results indicated a significant difference for suture by exposure interaction term, F = 7.215, p = 0.011 with PCNA increasing with citalopram exposure for the sagittal suture and the coronal not changing or slightly decreasing. Two-way analyses of variance were conducted for the coronal and sagittal sutures. The results for the coronal suture are illustrated in Fig 1C. Data suggest slight decreases in markers of proliferation in the periosteal and midsutural areas analyzed. The assumption of homogeneity of variance was met. Statistical analyses revealed no significant differences for exposure by area, or exposure and area main effects, p>0.05. The results for the sagittal suture are illustrated in Fig 1F. Data suggest increases in all suture areas analyzed. Statistical analyses revealed a significant difference by exposure, F = 6.203, p = 0.024, with the SSRI exposed sutures demonstrating greater PCNA positivity (47.94+/-35.47 > 21.38+/-16.15).

**Fig 2:**
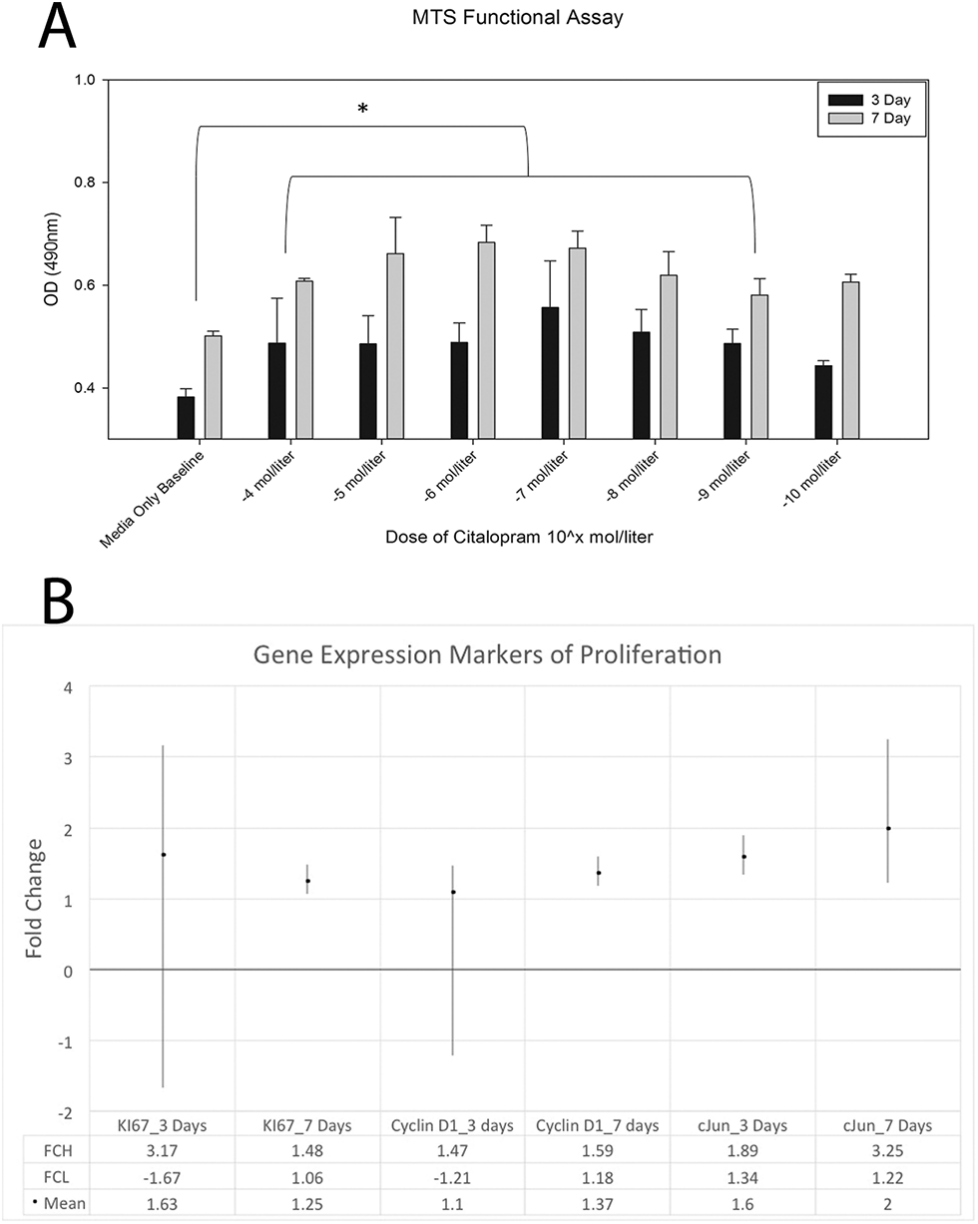
Assessment of *in vitro* markers of proliferation after citalopram treatment. A. MTS functional assay results from MC3T3 cells treated with serial dilutions of citalopram (10^−4^ mol through 10^−10^ mol). Significant time and dose effects are noted. B. Gene expression markers of proliferation from MC3T3 cells treated with media only or media supplemented with 10^−4^ mol citalopram. All three markers indicate a biological change due to treatment by 7 days. *Jun* was found to have significantly greater expression due to treatment at both 3 and 7 days.

Fig 2A demonstrates results for analysis of proliferation via MTS assay for MC3T3-E1 cells after citalopram treatment in culture for 3 and 7 days. Qualitative assessment suggests an increase in MTS activity for all treated groups compared to untreated baseline control. A two way analysis of variance was conducted for days in culture by citalopram dose for MTS activity. Assumptions of normality and homogeneity of variance were met. Results suggest significant differences in MTS activity by day, F = 105.062, p<0.001, with greater activity observed after 7 days (0.62 +/-0.06) compared to 3 days (0.48 +/-0.07). There was also a significant effect by dose F = 7.515, p<0.001. Post-hoc Bonferonni analysis revealed media only treatment to result in significantly less MTS activity compared with all treated groups respectively, p<0.05, with the exception of 10^−10^ mol/liter. Fig 2B demonstrates qrt-PCR results for three markers of proliferation (*Ki67*, *Ccnd1*, and *Jun*) after 3 and 7 days of citalopram treatment at 10^−4^ mol/liter on MC3T3-E1 cells. Results suggest mean increases for all markers at both time points. After 7 days all three markers are found to have confidence intervals that do not cross 1 indicating purported biological change. *Jun* is found to be significantly different between treatment and control at both 3 and 7 days.

### Markers of Apoptosis

**Fig 3:**
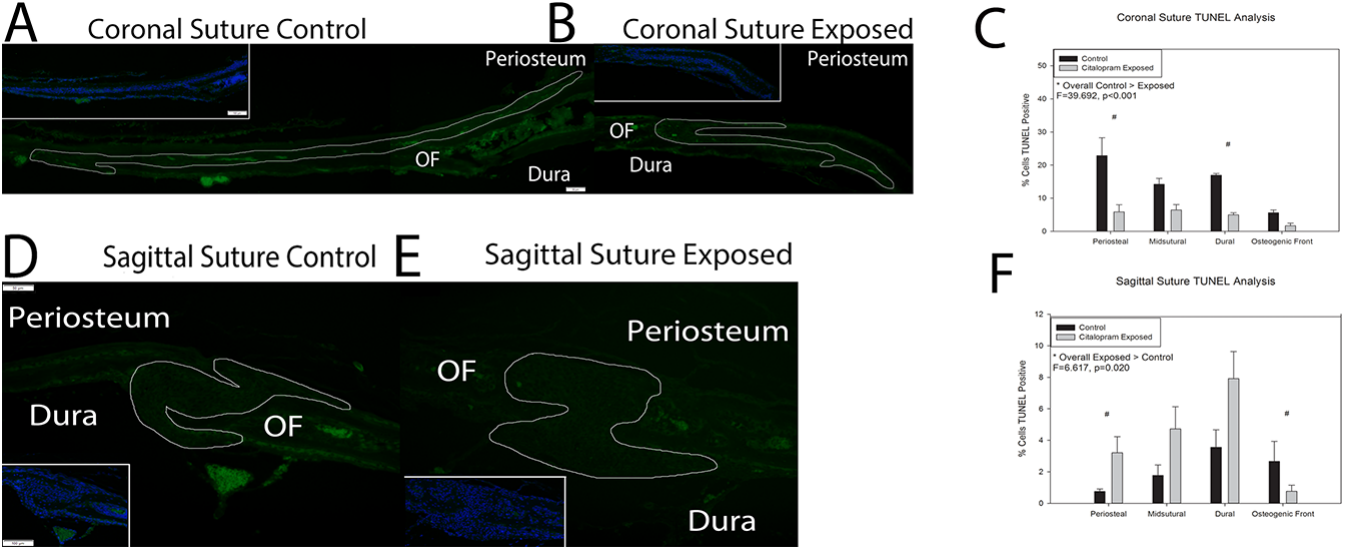
Histological markers of apoptosis in control and citalopram exposed mouse cranial sutures. A & B. Coronal sutures from control (A) and citalopram exposed (B) 15 day old C57BL6 mice stained for Terminal deoxynucleotidyl transferase dUTP nick end labeling (TUNEL) activity (green). Periosteum, dura and osteogenic front (OF) are as marked, and the sutural area has been outlined. Inset lower magnification of TUNEL staining with a 4',6-diamidino-2-phenylindole (DAPI) nuclear counterstain (blue). Note the highly cellularized (blue stained) area of the suture abutting the less cellularized (darker) area of the osteogenic front. C. Quantification of percent TUNEL positive staining as compared to DAPI stain in coronal sutures. Significant decreases in TUNEL activity were noted due to citalopram exposure at the periosteal and dural areas of the sutures. Midsutral space and osteogenic fronts did not demonstrate significantly different staining between unexposed and citalopram exposed sutures. D & E. Sagittal sutures from unexposed (control) (D) and citalopram exposed (E) 15 day old C57BL6 mice stained for TUNEL activity (green). Inset lower magnification of TUNEL staining with a DAPI nuclear counterstain (blue) F. Quantification of percent TUNEL positive staining as compared to DAPI stain. A significant increase in TUNEL staining was noted in the periosteal area while a significant decrease in staining was noted at the osteogenic front of citalopram exposed sagittal sutures.

Fig 3A, 3B, 3D and 3E demonstrates representative samples of Terminal deoxynucleotidyl tranferase dUTP nick end labeling (TUNEL a method for detecting DNA fragmentation due to apoptosis) staining for coronal and sagittal sutures from postnatal day 15 animals either unexposed (control) or exposed to citalopram *in utero*. Qualitative assessment suggests decreased TUNEL staining for SSRI exposed coronal sutures. Sagittal sutures demonstrated very little TUNEL activity. Quantitative analysis was conducted for percent cells TUNEL positive as a function of total cells present. A three-way analysis of variance was conducted to determine if the relationship for exposure (control or citalopram) by area of suture was the same for coronal and sagittal suture samples. Assumptions of normality was met for all groups with the exception of osteogenic front coronal suture not exposed (control) and sagittal suture exposed to citalopram. ANOVA is robust against this violation. The assumption of homogeneity of variance was met. Results indicated a significant difference for suture by exposure interaction term, F = 46.169, p<0.001 with TUNEL decreasing with exposure for the coronal suture and the sagittal not changing or a slightly increasing. Two-way analyses of variance were conducted for coronal and sagittal suture. The results for the coronal suture are illustrated in Fig 3C. Data suggests decreases in markers of apoptosis in all areas analyzed. The assumption of homogeneity was met. Statistical analyses revealed significant differences for exposure F = 39.692, p<0.001 with SSRI exposed sutures (4.74+/-2.87) having statistically less TUNEL activity than control (14.90 +/- 7.76). There was also a significant difference by area F = 7.740, p = 0.02. Post-hoc bonferonni analyses revealed the osteogenic front to have significantly less TUNEL activity than the periosteal or dural area, p<0.05. The results for the sagittal suture are illustrated in Fig 2F. Data suggests very little TUNEL activity for the sagittal suture. The assumption of homogeneity was met. Statistical analyses revealed significant differences for exposure F = 6.617, p = 0.02 with SSRI exposed sutures (4.16+/-3.26) having statistically greater TUNEL activity than control (2.19 +/- 1.72). There was also a significant difference by area F = 5.775, p = 0.007. Post-hoc bonferonni analyses revealed the dural area to have significantly greater TUNEL activity than the periosteal or osteogenic front areas, p<0.05.

**Fig 4:**
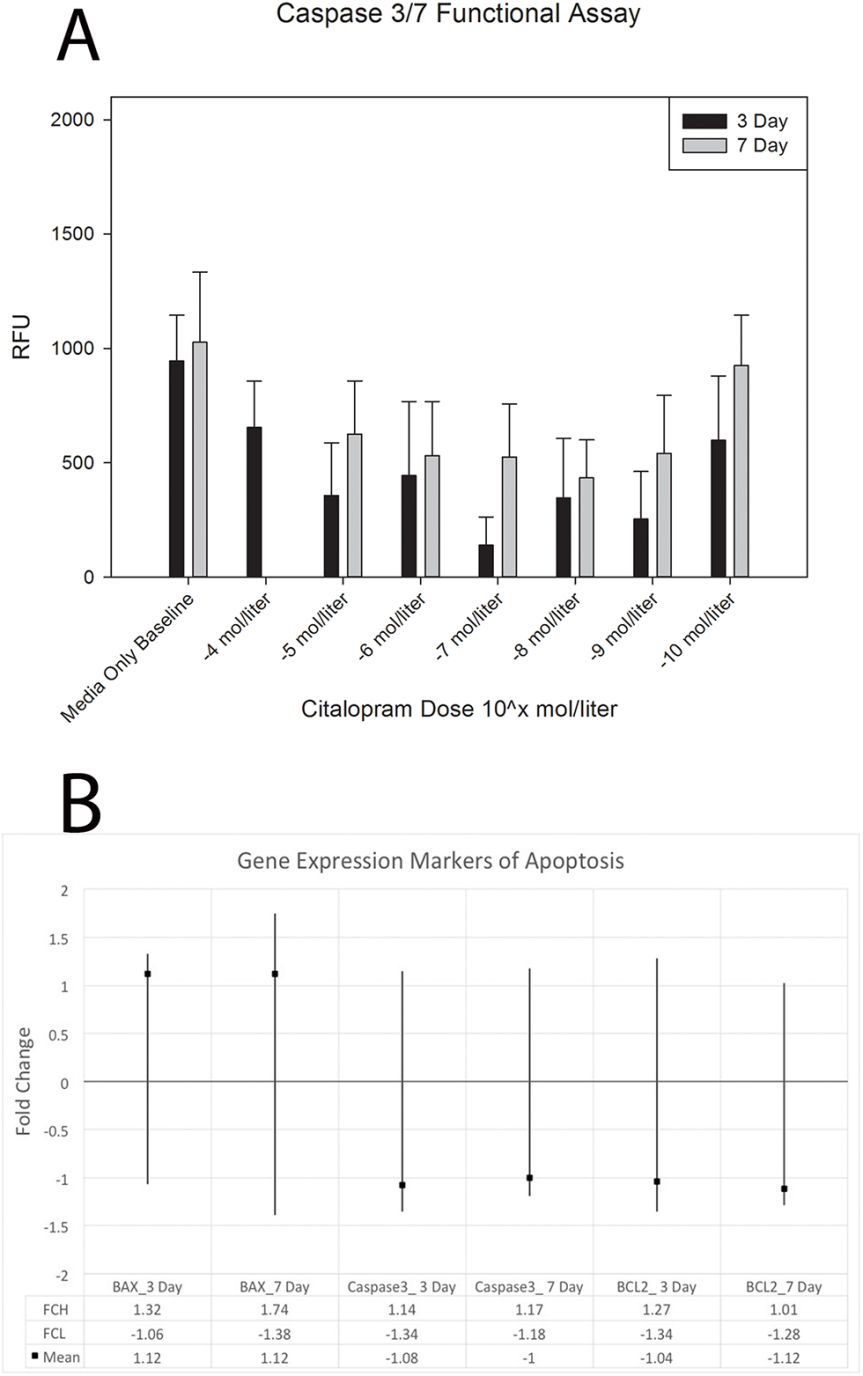
Assessment of *in vitro* markers of apoptosis after citalopram treatment. A. Caspase 3/7 functional assay results from MC3T3 cells treated with serial dilutions of citalopram (10^−4^ mol through 10^−10^ mol). No significantly significant differences were found by day, dose, or interaction terms. B. Gene expression markers of apoptosis from MC3T3 cells treated with media only or media supplemented with 10^−4^ mol citalopram. There were no statistically significant gene expression changes.

Fig 4A demonstrates results for apoptosis via Capase 3/7 activity analysis of MC3T3-E1 cells after citalopram treatment in culture for 3 and 7 days. Qualitative assessment suggests decreases in caspase activity for all treated groups compared to untreated baseline control. In addition no caspase activity was detected for the 10^−4^ mol/liter after 7 days in culture. A two way analysis of variance was conducted for days in culture by citalopram dose for Caspase activity. Assumptions of normality and homogeneity of variance were met. Results suggest no statistically significant differences in Caspase activity by day, dose or interaction terms. Fig 4B demonstrates qrt-PCR results for three markers of apoptosis (*Bax*, *Bcl2*, and *Casp3*) after 3 and 7 days of citalopram treatment at 10^−4^ mol/liter on MC3T3-E1 cells. All markers had confidence intervals crossing 1 and mean fold changes around 1 suggesting no biological changes. *Bax* was slightly above 1 fold change suggesting slight increase for both time points and *Casp3* and *Bcl2* showed slight decreases for both time points. There were no statistically significant gene expression changes.

## Discussion

Here we set out to determine if exposure to SSRIs would alter the cell cycle (proliferation and apoptosis) of perisutural cells at the cellular and transcription levels. Our previous data suggested that there was some mechanism causing measurable growth alterations even when dosages of SSRI were moderate to low. We sought to determine if there was a specific effect of SSRI treatment on perisutural cell cycle regulation disrupting homeostasis [24]. For proliferation measured in the sutural areas of mice exposed to the SSRI citalopram, there seems to be little exposure related effect in the coronal suture, whereas there is a greater effect in the sagittal suture due to exposure. The effect of increasing PCNA (a marker for proliferation) in suture associated cells was confirmed with the increase in MTS activity in murine pre-osteoblast cells over time and dosage of SSRI. TUNEL staining for apoptotic activity indicated that there was a relative decrease in apoptotic activity in coronal sutures exposed to SSRI as compared to control and a similar result was shown for the sagittal suture with the exception of an increase in apoptotic activity due to SSRI exposure recorded at the periosteal end of the suture. Minimal changes in apoptotic activity of cells treated with citalopram and in apoptosis related gene expression correlate with the very small changes demonstrated in the histomorphometric analysis.

It appears that overall exposure to the SSRI citalopram has a greater effect on proliferation and a lesser effect on apoptotic activity. Together these changes indicate that exposure to SSRIs affects cell cycle regulation by increasing proliferative potential and decreasing apoptotic activity in a site specific manner that may be indicative of hyperplasia. By analyzing the proliferative and apoptotic activity of control and citalopram exposed sutures we were able to identify site specific cell cycle changes that may have been missed if the suture has been analyzed as a whole. The decrease in TUNEL staining, indicating a decrease in apoptotic activity seen at the dural edge of the coronal suture in conjunction with the small increase in PCNA staining indicating an increase in proliferation in the same area may be indicative of hyperplasia. Likewise, the increase in TUNEL staining at the periosteum associated edge of the sagittal suture in conjunction with the increase in PCNA activity in that same area still results in a suture with relatively more cells at the dural edge than the periosteal edge. Such greater cell division and decreased programmed cell death may have specific implications for suture development. Hyperplasia may likely result in hyperostosis at the suture possibly leading to obliteration and synostosis. Research has indicated suture fusion often begins endocranially (near the dura) [41]. This is significant as our data suggests a pattern with increased cell cycle dysregulation near the dura which may lead to premature suture fusion.

Likewise, the assessment of genetic markers of cell cycle regulation and the balance between proliferation and apoptosis point towards a situation of hyperplasia resulting from exposure to SSRIs. The genetic markers of cell cycle progression were upregulated in suture associated cells when exposed to citalopram. Increasing expression of *Ki67*, *Ccnd1* and its regulating gene *Jun* all indicate an increase in cell cycle progression due to exposure to SSRI. The initial increase in *Jun* expression due to SSRI exposure is predictably followed by increased expression of *Ccnd1* indicating more cells in the G1 phase of the cell cycle. This increase in proliferative activity due to SSRI exposure is confirmed by an increase in *Ki67* expression indicating that cells are progressing through all phases of the cell cycle [42, 43]. Together these genes indicate an increase in cell cycling and thus proliferation. Additionally, upregulation of *Jun* is also indicative of anti-apoptotic activity which may be balancing the lack of pro-survival gene *Bcl2* expression seen in the perisutural cells. Though there may be a slight decrease in expression of *Bcl2* in response to treatment with the SSRI citalopram there is no associated increase in expression of *Bax* and therefore there is also no increase in expression of *Casp3* to facilitate apoptotic activity [42]. Thus, though there is no change in genes associated with apoptosis recorded here, the increase in proliferation related gene expression observed is likely sufficient to cause an imbalance in cell cycle regulation. Overall it seems as if there is a disruption in the balance of proliferative and apoptotic activity that results from exposure to citalopram.

The tight coordination of cellular proliferation and differentiation are known to be important for maintenance of the patent cranial suture and the eventual or pathological fusion. These balances are often cited as being controlled by regulatory networks known to be involved in craniofacial growth and development, including *MSX*, *FGF*s, *TGFβ*s and *TWIST* genes [27–31, 44–50]. We have previously shown that citalopram alters FGF, MSX and TGFB expression in osteoblast cell culture [24]. In addition, several preclinical studies have also suggested apoptosis to be necessary for the maintenance of the suture [25, 26]. Here we found that exposure to the SSRI citalopram was able to significantly alter the balance of cell proliferation and differentiation within the suture. Here these alterations were identified in a wild-type model not genetically predisposed to craniofacial abnormalities such as craniosynostosis, as we would predict in those with certain gene mutations citalopram exposure may exacerbate dysfunction.

As most cases of craniosynostosis are non-syndromic with no identified genetic mutation efforts are now focusing on the association of potential mediators on calvarial growth and development and synostosis [51, 52]. SSRI use (exposure to fetus through blood/placental barrier) has been identified as having an elevated risk association for craniosynostosis [1, 2, 4, 5, 18, 19]. Evidence here suggests these drugs, even at low and variable dosages, may have a direct effect on the homeostasis of cells within the calvaria and suture. Importantly, the differential effect by area seen here could be indicative of cell type specific effects in the suture areas defined by consisting of multiple cell types. A future assessment of the effect of citalopram exposure on heterogeneous cell type primary cells derived from sutures would be appropriate. Future research should expand on modeling these SSRIs for dose by time interaction to study craniofacial growth and identify potential caustic doses or windows of susceptibility effects on development.
